# Comprehensive analysis of transcriptomics and metabolomics provides insights into the mechanism by plant growth regulators affect the quality of jujube (*Ziziphus jujuba* Mill.) fruit

**DOI:** 10.1371/journal.pone.0305185

**Published:** 2024-08-23

**Authors:** Defen Liu, Na Jiang, Yuting Yuan, Hejiang Liu, Yanjun Ju, Wanjin Sun, Wenzhao Jia, Yi Fang, Duoyong Zhao, Jiefei Mao, Lu Kang

**Affiliations:** 1 Xinjiang Academy of Agricultural Sciences Institute of Agricultural Quality Standards and Testing Technology/Xinjiang Key Laboratory of Agricultural Product Quality and Safety, Urumqi, China; 2 Key Laboratory of Ecological Safety and Sustainable Development in Arid Lands, Xinjiang Institute of Ecology and Geography, State Key Laboratory of Desert and Oasis Ecology, Chinese Academy of Sciences, Urumqi, China; 3 Social Affairs Service Center of the Eighth Regiment of the First Division of Xinjiang Production and Construction Corps, Alar, China; 4 Agricultural Development Service Center of the Eighth Regiment of the First Division of Xinjiang Production and Construction Corps, Alar, China; 5 College of Food Science and Pharmacy, Xinjiang Agricultural University, Urumqi, China; Shiraz University, ISLAMIC REPUBLIC OF IRAN

## Abstract

A comprehensively analysis of the transcriptomics and metabolomics was conducted to investigate the mechanism of plant growth regulators on the quality of jujube fruit. After the application of plant growth regulators, a total of 3097 differentially expressed genes (DEGs) were identified, which were mainly annotated in 123 pathways such as flavonoid biosynthesis, metabolism of alanine, aspartate, and glutamate. In addition, 1091 differential expressed metabolites (DEMs), including 519 up-regulated and 572 down-regulated metabolites, were significantly altered after application of plant growth regulators. DEGs and DEMs simultaneously annotated 69 metabolic pathways, including biosynthesis of phenylpropane, flavonoid, starch and sucrose. The key genes in flavonoid biosynthesis pathway were revealed, which may play an important role in plant growth regulator regulation quality of jujube fruit. Besides, the application of plant growth regulator during the jujube flowering period increased the contents of gibberellin and indole-3-acetic acid in leaves, and decreased the contents of abscisic acid. The results may help to reveal the metabolic network and molecular mechanism of plant growth regulators in jujube fruit.

## 1. Introdcution

Jujube, also known as red date (*Ziziphus jujuba* Mill.) is a small deciduous tree. Jujubes originated in Asia and have been cultivated in China for more than 4000 years [1], leading to the selection of over 700 cultivars. China is the only exporter of jujube and accounts for over 90% of the world’s total production [2]. In China, jujube cultivation is mainly distributed in five provinces in the middle and lower reaches of the Yellow River and the Aksu, Hotan, Kashgar and Hami of Xinjiang. According to the Chinese National Bureau of Statistics [3] and Statistical Yearbook of Xinjiang [4], the planting area of jujube trees in China in 2020 was approximately 3.3 million hectares, with a fruit yield of 8.8 million tons. In Xinjiang, the planting area was 414,000 hectares, with a yield of 3.8 million tons, accounting for 43.4% of the national production. The jujube industry is a characteristics and important pillar industry for agricultural development in southern Xinjiang, providing strong support for local growers to increase their income and playing a crucial role in promoting economic development [5,6].

Jujube are rich in nutrients, including polysaccharides [7], flavonoids [8], polyphenols [9–11], cyclic adenosine monophosphate (cAMP) [12], organic acids, and saponins [13]. They are also considered to assist in oxygen free radical scavenging, cardiovascular regulation, anti-aging, cancer prevention and to enhance immune function. Jujube trees display indefinite inflorescence with a long flowering period, during the growth and development of jujube fruit in most cultivars, this is accompanied by a low natural fruit setting rate due to abscisic acid [1,14,15]. The main jujube cultivar in southern Xinjiang, jujube has a natural fruit setting rate of only about 1% of the total number of flowers [14]. Gibberellic acid (GA_3_) is often sprayed during the flowering period of jujube trees, which can improve fruit setting by up to 33% [16]. The effect of increasing yield is most significant when spraying a concentration of 40 mg/L GA_3_ during the peak flowering period of jujube [17]. The application of plant growth regulators can regulate the growth and quality of plant fruits [18,19]. GA_3_ and other gibberellins (GAs) has been reported to promote seed germination, plant growth, flowering and fruit ripening [20–22]. During these processes, GAs play an important role in the regulation of other endogenous phytohormones, especially during flowering [23].

GAs promote the growth and development of flower organs [24]. GA_3_ is the most commonly used plant growth promoter in the commercial sector [20,21]. Exogenous GA_3_ can differentially affect the content of other plant hormones in different plant species. In sugarcane and watermelon, the exogenous application of gibberellin caused significant increases in the endogenous hormones, indole-3-acetic acid (IAA) and cytokinin, while abscisic acid (ABA) was significantly decreased [25]. In contrast, exogenous GA_3_ treatment of jujube significantly increase the content of GA_3_, IAA and ABA [26]. In grapevine, the application of GA_3_ can promote fruit setting, grape expansion, coloring, development and ripening, thereby improving grape yields [27]. In addition, GA_3_ has been reported to improve the hardness, total carotenoids, and polyphenol content of cashew nuts [28]. GA_3_ can also increase cell flexibility and peel extension, thus reducing the cracking of many cultivated fruits [15,29]. However, plant growth regulators are considered to be over-applied in fruit tree cultivation [30,31] and can lead to decreased product quality. For example, treatments of jujube with GA_3_ resulted in a decrease in jujube soluble solids sugars [32]. The brassinosteroids (BRs) is steroid plant hormones that play a major role in regulating fruit ripening in various species. In apples, BR was involved in the biosynthesis pathway of volatile compounds in the fruit [33] and had also been applied in research on bananas [34]. Recent studies have shown that green soybean seeds treated with BR can improve the quality and subsequent physiological development of soybean seedlings [35], and BR also plays a role in regulating wheat flower degradation under low nitrogen stress [36].

Plant growth regulators regulate many plant metabolic processes [37]. The thidiazuron (TDZ) affects plant growth and development in low concentrations [38]. TDZ can promote grape berry enlargement and weight gain [39]. Treating grape inflorescences with 2.5 mg/L TDZ and 25 mg/L GA_3_ was also reported to improve the berry solid acid ratio [40]. In recent studies, TDZ has effectively improved the quality and size of kiwifruit, as well as the content of soluble solids, and reduced fruit hardness [41]. The TDZ significantly increased the size and weight of mango fruits, reduced the disease index, and maintained relatively high levels of hardness and antioxidant capacity during post-harvest storage [42]. RNA-Seq analysis indicated GA_3_ of grapes resulted mainly in the differential expression of genes involved in flavonoid and phenylpropanoid biosynthesis [43]. GA_3_ affects the regulation of phenylpropanoid synthesis and cell wall formation [44]. RNA-Seq analysis showed that GA_3_-induced cherry parthenogenesis resulted in significant enrichment of differentially expressed genes (DEGs) in phenylalanine metabolism and phenylpropanoid biosynthesis [45].

The GA_3_ of longan fruits was shown to up-regulate many tannins, phenolic acids and lignans during the on-tree preservation period [46]. The IAA treatment of kiwifruit resulted in the activation of the phenylpropanoid synthesis pathway, including flavonoids, phenols, terpenoids, as well as carbohydrate metabolism and hormone signaling pathways [47]. The present investigation utilized a comprehensive analysis of transcriptomics and metabolomics to comprehensively analyze the effect of spraying plant growth regulators on the quality of jujube fruit. The screening of candidate compounds and genes, as well as clarifying the metabolic regulatory network under the effects of GA_3_, BR, and TDZ. We hypothesized that plant growth regulators increased the fruit setting rate of jujube and related to the biosynthesis of flavonoids. The study results are of great significance for understanding how plant growth regulators improve the fruit setting rate and quality of jujube.

## 2. Materials and methods

### 2.1. Experimental design

The field trials of jujube were conducted in the 8^th^ regiment, Alar city, Xinjiang at May 30, 2022 during the jujube blooming period. The six experimental treatments were applied twice by spray with 7 days interval and consisted of J1 (18 g·hm^−2^ GA_3_), J2 (18 g·hm^−2^ GA_3_ and 45 mg·hm^−2^ BR), J3 (18 g·hm^−2^ GA_3_, 45 mg·hm^−2^ BR and 1.8 mg·hm^−2^ TDZ), J4 (36 g·hm^−2^ GA_3_), J5 (36 g·hm^−2^ GA_3_ and 45 mg·hm^−2^ BR), and J6 (36 g·hm^−2^ GA_3_, 45 mg·hm^−2^ BR and 1.8 mg·hm^−2^ TDZ). Spraying water were as the control group (CK). The leaves were collected a 2 h, 1 d, 3 d, 5 d, 7 d and 14 d after the completion of each treatment. The mature fruits were collected in late September 2022. In each case, three replicates were collected, immediately frozen in liquid nitrogen, placed in dry ice for transport and stored at −80°C until analysis.

### 2.2. The effects of plant growth regulator on quality of jujube fruit

#### 2.2.1. Ultra performance liquid chromatography and tandem mass spectrometry (UPLC-MS/MS) analysis of phytohormones, flavonoids and polyphenols

The extraction of phytohormones was performed as described [24]. Flavonoids and polyphenols were extracted using a modification of a reported method [48]. Briefly, 0.5 g of the sample was sonicated (40 kHz, 25°C) in 4 mL of acetonitrile for 30 minutes, then clarified by centrifugation at 5000 *g* and filtered through a 0.22 μm filter. The flavonoid and polyphenol contents were determined using a UPLC-MS/MS (Waters, Milford, MA, USA) with reference to standards (S1 Table). The UPLC utilized an Acquity UPLC C18 column (2.1 × 100 mm, 1.7 μm; Waters, Milford, MA, USA). For the separation of phytohormones (5 μL injection volume), the column temperature was set at 40°C. The mobile phase consisted of acetonitrile (A) and water (B) with a flow rate of 0.25 mL/min within a gradient of 10% A, 0–3.0 min; 3.0–7.0 min, 10%–50% A, 3.0–7.0 min; 50%–98% A, 7.0–18.0 min. For the separation of flavonoids and polyphenols (10 μL injection volume, column temperature 30°C), the mobile phase consisted of methanol (A) and water (B) with a flow rate of 0.2 mL/min within a gradient consisting of 5% A, 0–3.0 min; 5–45% A, 3.0–7.0 min; 45–50% A, 7.0–7.5 min; 50–75% A, 7.5–9.0 min; 75–90% A, 9.0–13.0 min. The MS/MS conditions was set for multiple reaction monitoring mode (MRM) in the negative ion mode. The electron spray ionization gas temperature was set at 500°C, the ion spray voltage at −4500 V, the ion source temperature at 350°C and the de-solvent gas temperature at 650°C. The cone voltage, collision energy, quantitative and qualitative ion pair MS/MS parameters of each metabolite were shown in S2 Table.

#### 2.2.2. Determination of fructose, glucose and sucrose

The contents of fructose, glucose, sucrose, organic acid and cyclic adenosine phosphate were determined using a Waters UPLC e2695 separation module (Waters, Massachusetts, United States). The fruit contents of the soluble sugars fructose, glucose and sucrose were determined by UPLC according to GB 5009.8–2016 [49]. The chromatographic column used was a NH_2_-RP column (250 × 4.6 mm, 5 μm) (Macherey Nagel, California, United States) with a column temperature of 40°C. The mobile phase consists of 70% acetonitrile in water with a flow rate of 1.0 mL/min. Detection employed a Waters 2414 refractive index detector (Waters, MA, USA) at 40°C.

#### 2.2.3. Determination of organic acid

Organic acids were determined by HPLC according to GB 5009.157–2016 [50]. The column used was a ODS column (250 mm × 4.6 mm, 5μm) (Dima Technology, Shanghai, China). Column temperature: 40°C. The mobile phase consists of (A) 0.1% phosphoric acid solution and (B) methanol (Thermo Field, Shanghai, China). The flow rate was 1.0 mL/min; injection volume: 20 μL; the mobile phase A: B ratio was 97.5%: 2.5%. Metabolite detection used a Waters 2489 UV/Vis Detector (Waters, MA, USA) set at 210 nm.

#### 2.2.4. Determination of cyclic adenosine monophosphate (cAMP) and lignin

The cAMP was extracted as described by Zhao [51], and quantified by HPLC, using an Agilent Zorbax SB C18 column (4.6 mm × 250 mm, 5 μm) (Agilent, California, USA) with a column temperature of 35°C. The mobile phase was 15% methanol and 85% 50 mM potassium dihydrogen phosphate with a flow rate of 0.8 mL/min and an injection volume of 10 μL. The detection wavelength was set at 254 nm. The lignin content in samples was determined using a commercial assay kit (Solarbio, Beijing, China) as the manufacturer’s instructions.

#### 2.2.5. Determination of volatile compounds

The extraction for volatile compounds of jujube fruit according to He [52]. Take 10 g of powder sample, add 80, 50, and 50 mL of dichloromethane by extract three times, respectively. After shake for 30 minutes each time, filter and merge the organic phase. The organic phase was evaporated to dryness in a rotating evaporator at 40°C, and finally fixed volume with 1 mL of ethyl acetate. After passing through a 0.22 μm filter membrane, it was loaded into an injection vial for GC-MS determination.

### 2.3 Transcriptomics (RNA-Seq)

Three biological replicates of each treatment group were sequenced. Total RNA extraction and sequencing were undertaken by Metware Co., Ltd. (Wuhan, China) on the Illumina Hiseq sequencer platform (Illumina, CA, USA). Raw reads were filtered to remove containing adapter sequences and Poly-N (> 10%) reads. Retained reads were mapped to predicted jujube mRNA using Hisat 2.2.1 software [53] and the jujube reference genome obtained from the NCBI genome database (https://www.ncbi.nlm.nih.gov/genome). The levels of gene expression were expressed in fragments per kilobase of transcript per million mapped reads (FPKM). The DESeq2 (1.22.1) was detected DEGs for each pair of experimental conditions [54]. Genes displaying an adjusted p-value < 0.05 and a log_2_ Fold Change (FC) > 1 were considered DEGs. Functional annotations of the DEGs were performed on 7 different databases (S3 Table). Gene Ontology (GO) [55] enrichment analysis were performed using cluster profiler (V 4.6.0) software, while KEGG (Kyoto Encyclopedia of Genes and Genomes) signaling pathway enrichment analysis were performed using online KEGG resources (https://www.genome.jp/kegg). The GO terms and KEGG pathways enriched with a false discovery rate (FDR) ≤ 0.05 were selected.

### 2.4. Quantitative real‑time polymerase chain reaction (qRT-PCR) analysis

Total RNA was extracted using a total plant RNA extraction kit (Biospin, Hangzhou, China). The RNA quality was assayed using Nano-Photometer ®N60 instrument (IMPLE, Beijing, China). The total RNA was reverse-transcribed into cDNA using one-step gDNA removal and cDNA synthesis kit (Tran, Beijing, China) as per the manufacturer’s instructions. The qRT-PCR utilized the Light Cycler®96 instrument (Roche, Shanghai, China) using the primer sequences described in S4 Table and *ACT1* as the reference gene. The 2^−ΔΔCT^ method [56] was used to determine the relative mRNA levels.

### 2.5. Detection and analysis of a wide range of targeted metabolomes

#### 2.5.1. Metabolite extraction

The metabolomics were carried out by Metware Biotechnology Co., Ltd. Jujube fruit were vacuum freeze-dried then ground to powder. The 50 mg of the powder was added to 1.2 mL of 70% precooled methanol and maintained at −20°C with vortexed for 30 s every 30 min for a total of 6 times. The extract was then clarified by centrifugation at 13,400 *g* for 3 min and the supernatant was filtered with a 0.22 μm microporous filter membrane.

#### 2.5.2. UPLC-MS/MS conditions

The sample extracts were analyzed using an UPLC-MS/MS (UPLC, ExionLC™ AD) and Tandem mass spectrometry system (Sciex, MA, USA). The analytical conditions were as follows: an Agilent SB-C18 (2.1 × 100 mm, 1.8 μm) column with a column temperature of 40°C was used for metabolite separation. The mobile phase consisted of water (A) and acetonitrile (B), each containing 0.1% (v/v) formic acid. The elution gradient consisted of 5% B, 0–1 min; 5%−95% B, 1–9 min; 95% B, 9–10 min, with a flow rate of 0.35 mL/min. The sample injection volume was 2 μL. The MS/MS analysis of metabolites utilized MRM with optimization of the declustering potential and collision energy of each ion pair. The electrospray ionization (ESI) temperature used was 500°C and the ion spray voltages were set at 5500 V and -4500V in the positive and negative ion modes, respectively. The ion source gas I, II and curtain gas pressures were set at 50, 60 and 25 psi, respectively.

#### 2.5.3. Metabolite quantification and the identification of differentially expressed metabolites (DEMs)

The DEMs were accepted a significantly different when variable interdependent parameter (VIP) scores > 1 [57] and Log_2_FC ≥ 1.0 were achieved. VIP values were extracted from OPLS-DA data, which also contain score plots and permutation plots, and was generated using R package Metabo Analyst R (1.0.1). The data was Log transform (Log_2_) and mean centering before OPLS-DA. In order to avoid overfitting, a permutation test was performed.

### 2.6. Statistical analysis

The least significant difference (LSD) of ANOVA was calculated using IBM SPSS statistics 26.0 and used at 5% probability level.

## 3. Results

### 3.1. Effects of different plant growth regulators on jujube leaf phytohormone content

To gain insight into the effects of plant growth regulator treatments on phytohormone levels, phytohormones which have been closely associated with jujube fruit setting [58,59] were determined during the blooming period of flowering, including four GA (GA_1_, GA_3_, GA_4_ and GA_7_), IAA and ABA. The application of GA_3_, BR and TDZ to leaves had a positive impact on the jujube phytohormone levels tested. The contents of the GA_1_, GA_3_ were increased with increasing GA_3_ concentration, however, compared with CK, there was only a significant difference in the content of GA_4_ after J1 and J4 treatments at 5 d; the content of GA_7_ in leaves showed no significant difference at 3 d, 14 d after treatment with JI and J4. The content of GA_1_ and GA_3_ were the main GA. The effects of plant growth regulators on jujube plant hormones were shown in Table 1. After applying different plant growth regulators at 14 d, the content of GA_1_, IAA, and ABA in the leaves showed significant changes under all treatment groups. The content of GA_3_ did not change significantly at J3, while the content of GA_4_ and GA_7_ only showed significant differences at J2 and J5. The content of GA_7_ and GA_4_ at J4 increased significantly by 344.8% and 471.2% compared with CK at 5 d, respectively. The content of IAA increased by 104.9%, 90.8%, 154.6%, 270.2%, and 283.7% in J1, J2, J4, J5, and J6 treatment groups compared with the control group at 5 d, respectively. Conversely, the IAA content decreased by 26.0% at J3.

**Table 1:** Effects of different plant growth regulator formulations on jujube leaf hormone levels (ng·g^-1^).

Time		Item	CK	J1	J2	J3	J4	J5	J6
2 h	GA_1_		186.94±31.57^f^	535.31±34.20^e^	627.76±67.78^d^	858.61±42.80^c^	974.91±51.01^b^	1151.31±44.57^a^	818.18±41.06^c^
	GA_3_		75.23±14.41^f^	1138.37±56.80^e^	3148.98±69.07^b^	2071.88±80.14^c^	3084.28±91.21^b^	3604.38±93.25^a^	1510.42±18.60^d^
	GA_4_		0.16±0.12^e^	3.07±0.41^d^	5.41±0.20^c^	6.57±0.28^b^	2.82±0.36^d^	2.88±0.24^d^	7.30±0.39^a^
	GA_7_		0.43±0.04^f^	6.03±0.04^b^	7.96±0.19^a^	5.31±0.25^c^	3.60±0.26^d^	2.77±0.18^e^	5.89±0.15^b^
	IAA		1.16±0.05^c^	1.66±0.29^abc^	1.76±0.42^ab^	1.22±0.19^bc^	1.87±0.20^a^	1.20±0.49^bc^	1.41±0.18^abc^
	ABA		13.93±0.53^a^	5.24±0.67^c^	4.99±0.63^cd^	7.49±0.54^b^	3.61±1.37^cd^	4.17±1.87^cd^	3.11±0.65^d^
1 d	GA_1_		163.90±130.60^d^	1166.85±92.94^a^	949.40±59.38^bc^	1118.85±45.89^ab^	820.87±50.98^c^	951.38±158.18^bc^	1260.74±62.04^a^
	GA_3_		74.04±35.21^e^	1876.77±29.36^d^	2230.96±26.43^b^	2396.84±39.59^a^	1837.50±36.95^d^	2086.92±55.43^c^	2106.68±46.01^c^
	GA_4_		0.59±0.23^d^	0.76±0.03^d^	0.81±0.24^d^	1.18±0.33^bc^	0.89±0.04^cd^	1.40±0.23^b^	1.84±0.09^a^
	GA_7_		0.18±0.02^d^	0.27±0.22^d^	0.38±0.25^d^	0.44±0.30^d^	0.79±0.04^c^	1.70±0.04^b^	2.31±0.22^a^
	IAA		1.14±0.02^e^	4.31±0.60^c^	6.87±0.37^a^	3.66±0.34^cd^	3.87±0.12^c^	5.79±0.23^b^	3.09±0.63^d^
	ABA		10.41±0.41^a^	10.23±0.55^a^	3.38±0.33^cd^	4.62±0.54^b^	4.21±1.02^bc^	2.97±0.29^d^	2.60±0.40^d^
3 d	GA_1_		389.54±66.34^d^	495.86±102.36^d^	1253.34±39.23^a^	841.82±36.05^c^	826.18±54.81^c^	970.13±58.96^b^	1264.44±95.66^a^
	GA_3_		102.95±19.48^e^	333.50±81.76^cd^	937.70±66.83^a^	384.16±26.10^c^	269.18±27.54^d^	549.32±48.13^b^	616.71±27.97^b^
	GA_4_		0.20±0.05^c^	0.09±0.05^c^	0.76±0.06^a^	0.35±0.21^bc^	0.16±0.04^c^	0.51±0.27^ab^	0.24±0.14^bc^
	GA_7_		0.19±0.02^c^	0.52±0.22^c^	2.64±0.32^a^	0.35±0.29^c^	0.25±0.19^c^	0.93±0.05^b^	0.32±0.29^c^
	IAA		1.44±0.13^d^	3.84±0.16^b^	6.73±0.38^a^	2.15±0.30^c^	1.18±0.29^d^	2.60±0.45^c^	2.43±0.49^c^
	ABA		25.32±0.59^a^	16.06±0.20^b^	4.41±0.14^c^	2.98±0.62^d^	2.34±0.54^de^	1.60±0.33^e^	2.11±0.46^e^
5 d	GA_1_		483.21±46.95^f^	935.80±47.99^e^	1611.91±30.37^b^	1071.52±80.46^d^	1189.71±56.05^c^	2028.72±76.90^a^	1277.99±76.43^c^
	GA_3_		143.17±50.30^d^	287.64±69.47^cd^	910.41±85.28^a^	220.28±99.21^d^	463.23±37.33^b^	970.98±129.10^a^	415.67±84.23^bc^
	GA_4_		0.25±0.05^d^	0.35±0.13^cd^	0.68±0.28^bc^	0.78±0.19^b^	1.42±0.27^a^	0.60±0.03^bc^	0.61±0.05^bc^
	GA_7_		0.43±0.03^d^	0.47±0.28^d^	1.29±0.11^b^	1.00±0.05^c^	1.93±0.11^a^	0.76±0.23^c^	0.82±0.15^c^
	IAA		1.41±0.09^d^	2.89±0.43^bc^	2.69±0.26735^c^	1.043±0.31^d^	3.59±0.45^b^	5.22±0.43^a^	5.41±0.63^a^
	ABA		28.63±0.40^a^	10.07±0.38^b^	4.23±0.13^d^	4.99±0.09^c^	4.21±0.17^d^	3.08±0.12^e^	3.33±0.35^e^
7 d	GA_1_		575.64±16.97^c^	937.95±126.29^b^	894.36±92.57^b^	635.10±56.60^c^	1157.22±48.81^a^	718.41±89.08^c^	641.66±62.41^c^
	GA_3_		83.88±13.52^d^	273.22±119.70^b^	229.99±32.97^bc^	223.76±43.41^bc^	463.10±70.25^a^	325.91±90.05^b^	118.52±33.83^cd^
	GA_4_		0.26±0.04^a^	0.36±0.20^a^	0.40±0.14^a^	0.66±0.10^a^	0.37±0.27^a^	0.43±0.24^a^	0.58±0.39^a^
	GA_7_		0.63±0.06^c^	0.80±0.08^bc^	1.39±0.30^a^	1.02±0.21^b^	0.81±0.01^bc^	1.45±0.31^a^	0.68±0.19^bc^
	IAA		1.04±0.09^f^	3.34±0.21^e^	4.90±0.45^d^	6.44±0.37^b^	5.77±0.20^c^	6.14±0.13^bc^	8.89±0.19^a^
	ABA		19.21±0.46^a^	17.08±0.51^b^	5.36±1.10^c^	3.91±0.19^d^	2.86±0.80^e^	2.56±0.29^e^	2.12±0.07^e^
14 d	GA_1_		140.66±58.22^e^	638.40±32.98^b^	985.41±86.75^a^	250.30±25.03^d^	379.95±97.16^c^	423.09±52.79^c^	621.08±23.34^b^
	GA_3_		37.85±7.67^d^	98.67±30.83^c^	159.44±10.38^b^	40.04±2.51^d^	213.57±70.01^ab^	249.37±21.71^a^	46.98±22.89^cd^
	GA_4_		0.14±0.10^b^	0.07±0.03^b^	1.26±0.29^a^	0.18±0.09^b^	0.27±0.18^b^	0.99±0.19^a^	0.04±0.02^b^
	GA_7_		0.12±0.01^c^	0.20±0.07^c^	1.62±0.28^a^	0.19±0.09^c^	0.19±0.11^c^	1.20±0.22^b^	0.13±0.10^c^
	IAA		1.61±0.10^f^	5.63±0.32^e^	7.90±0.74^cd^	10.72±0.62^b^	7.02±0.35^d^	9.73±0.52^bc^	13.76±0.52^a^
	ABA		5.20±1.14^a^	4.71±0.80^ab^	3.59±0.49^bc^	3.23±0.63^c^	3.15±0.71^c^	1.39±0.33^d^	1.10±0.42^d^

Note: Different lowercase letters in each row indicate significant differences (*P*<0.05) between the plant growth regulator treatments. CK: Control group; J1: 18 g·hm^-2^ GA_3_; J2: 18 g·hm^-2^ GA_3_ and 45 mg·hm^-2^ BR; J3: 18 g·hm^-2^ GA_3_, 45 mg·hm^-2^ BR and 1.8 mg·hm^-2^ TDZ; J4: 36 g·hm^-2^ GA_3_; J5: 36 g·hm^-2^ GA_3_ and 45 mg·hm^-2^ BR; J6: 36 g·hm^-2^ GA_3_, 45 mg·hm^-2^ BR and 1.8 mg·hm^-2^ TDZ.

The ABA of all treatment groups were significantly lower than CK at 7−14 d. The GA_3_ content of J1 and J4 in the leaves increased and then rapidly decreased at 1 d. However, the GA_1_ content decreased at 3 d, but showed an increasing by BR at 5 d. Within 0−14 days, the IAA content of J1, J2, J4, J5, and J6 significantly increased and more significant by TDZ at 7−14 d. The IAA content of J3 decreased at 5 d. All treatment groups showed inhibited ABA content. In summary, the content of IAA and GA were increased at J4 and J6, but resulted in a decreased ABA.

### 3.2. Effects of different plant growth regulators on indicators of jujube fruit quality

**Fig 1:**
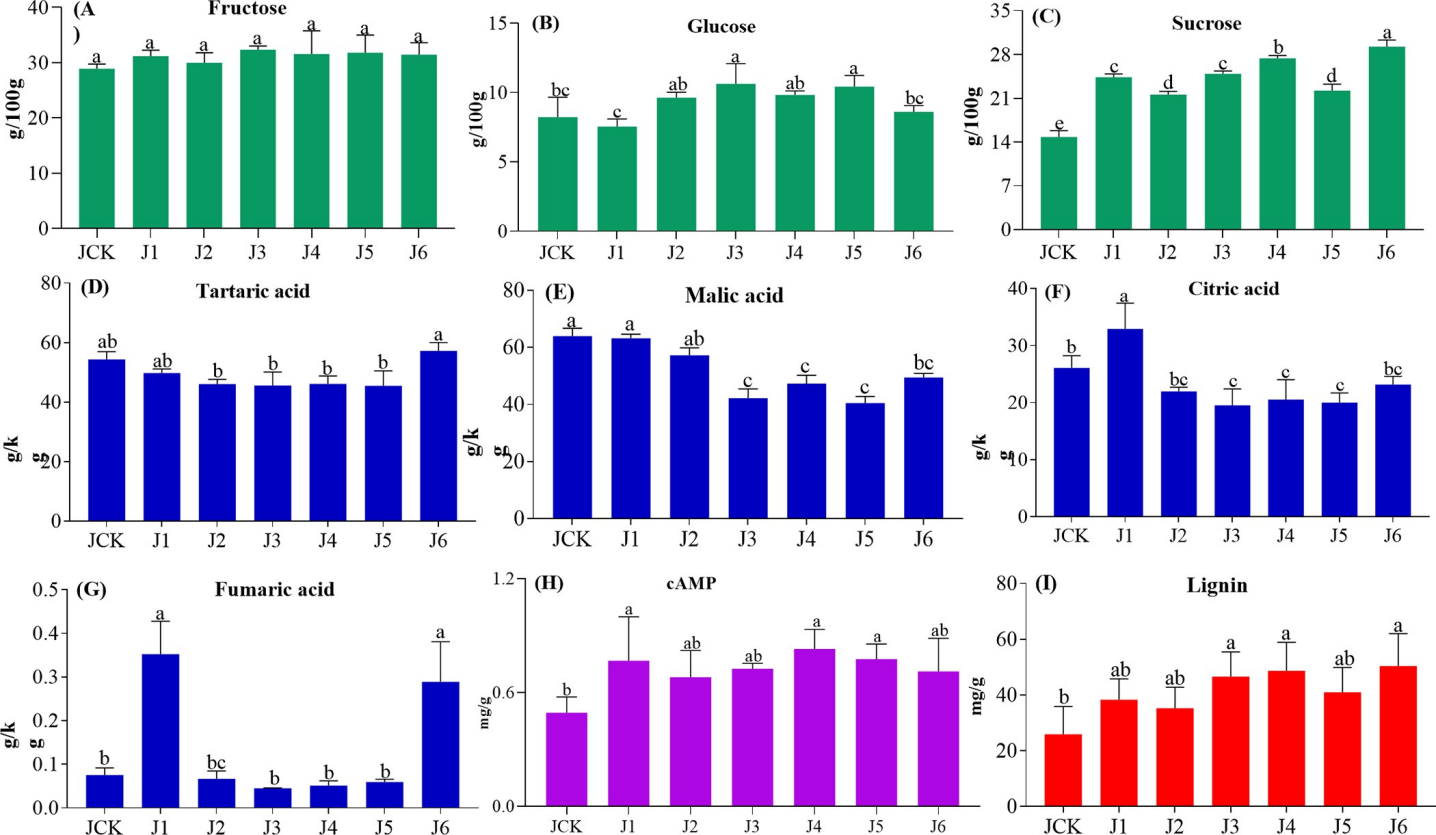
The effect of different plant growth regulator formulations on sugars, organic acid, cAMP and lignin of jujube fruit. **(**A-C) soluble sugars. (D-G) organic acids. (H) cyclic adenosine monophosphate (cAMP). (I) lignin. CK: Control; J1: 18 g·hm^-2^ GA_3_; J2: 18 g·hm^-2^ GA_3_ and 45 mg·hm^-2^ BR; J3: 18 g·hm^-2^ GA_3_, 45 mg·hm^-2^ BR and 1.8 mg·hm^-2^ TDZ; J4: 36 g·hm^-2^ GA_3_; J5: 36 g·hm^-2^ GA_3_ and 45 mg·hm^-2^ BR; J6: 36 g·hm^-2^ GA_3_, 45 mg·hm^-2^ BR and 1.8 mg·hm^-2^ TDZ. Different letters indicate a significant difference between treatments (*p*<0.05) and same letters indicate an insignificant difference between treatments (*p*≥0.05).

The effect of plant growth regulators on the transverse and longitudinal diameters of jujube are shown in S5 Table. The transverse diameters followed the order of J6 (5.6 cm) > J1 | J3 | J4 | J5 (5.0 cm) > CK | J2 (4.2 cm), respectively. The significant effects on the longitudinal diameter were only observed at J4. Together, the results indicate that the largest significant effects on fruit size was obtained at J4 and J6. The effects of plant growth regulators on soluble sugars are shown in Fig 1A–1C, which indicates that fructose levels were largely unaffected by the plant growth regulator. The minor effects on glucose levels were observed, which nevertheless achieved significance at J3 and J5. The larger relative changes were observed in sucrose levels, which follow the trend J6 > J4 > J1 | J3 > J2 | J5 > CK. The different plant hormone treatments had varying effects on jujube organic acids contents, except for tartaric acid, which was not significantly affected (Fig 1D–1G). The effects on fumaric acid and citric acid were moderate. The citric acid significantly increased at J1, while it decreased significantly at J3, J4, and J5. The malic acid also showed significant decreases at J3, J4, J5, and J6.

The cAMP content was increased at J1, J4, and J5 (Fig 1H). Similarly, higher lignin contents were observed after all treatment groups, with significant increases observed at J3, J4 and J6 (Fig 1I). The contents of hesperidin, catechin and rutin significantly increased, whereas the contents of naringenin, kaempferol and quercitin were not significantly affected (Table 2). Conversely, the contents of caffeic acid and ferulic acid in fruit were reduced (Table 3). The largest significant effects on the indicators could be seen at J4 and J6 relative to CK. The lignin, cAMP, and sucrose of J4 were significantly increased compared to CK, with decreases malic (26.0%), and citric acids (21.3%). The contents of lignin (94.2%), cAMP (44.1%), sucrose (97.4%) and fumaric acid (9.4%) of J6 significantly increased, with decreases in malic (22.7%), citric acids (11.3%). Therefore, the J4 and J6 were selected for further analysis. The effect of GA_3_ on the types of volatile compounds in jujube fruit are shown in S6 Table. The 6 categories and 50 volatile compounds were detected at CK, J1, and J4, with the highest number of acids, followed by esters, alcohols, and ketones. The number of ethers, aldehydes, and other volatile compounds was relatively small. The CK and treatment groups of jujube fruit contain 9 acids, 2 ketones, and 1 alcohol. The content of lauric acid and myristic acid is above 1%.

**Table 2:** UPLC analysis of plant growth regulator effects on flavonoid of jujube fruit (μg·g^-1^).

Treatment	Hesperidin	Catechin	Rutin	Naringenin	Kaempferol	Quercitin
CK	1.08±0.288^c^	0.59±0.09^d^	4.71±0.23e	-	-	-
J1	1.38±0.30b^c^	0.75±0.07^d^	5.81±0.50^d^	-	0.02±0.01^ab^	0.03±0.01^a^
J2	2.91±1.90^ab^	1.20±0.35^cd^	7.73±0.19^c^	0.09±0.01^a^	0.04±0.01^ab^	0.06±0.03^a^
J3	3.26±1.05^a^	1.73±0.16^bc^	8.03±0.35^c^	0.06±0.02^a^	0.05±0.01^a^	0.06±0.05^a^
J4	2.24±0.48^abc^	1.24±0.19^cd^	10.39±0.28^b^	0.05±0.01^ab^	0.03±0.01^ab^	0.06±0.01^a^
J5	4.01±0.73^a^	2.36±0.88^ab^	11.03±0.67^b^	0.06±0.05^a^	0.04±0.02^a^	0.07±0.02^a^
J6	3.03±0.87^ab^	2.48±0.24^a^	12.29±0.49^a^	0.08±0.02^a^	0.04±0.01^a^	-

Note: The lowercase letters, CK, J1, J2, J3, J4, J5 and J6 are as in Table 1.

**Table 3:** UPLC analysis of plant growth regulator effects on phenolic acid of jujube fruit (μg·g^-1^).

Treatment	Quinic acid	Chlorogenic acid	Caffeic acid	Ferulic acid	Trans-4-hydroxy-cinnamic acid
CK	20.43±3.28^d^	-	0.06±0.01^a^	0.05±0.01^a^	0.18±0.02^ab^
J1	30.88±2.68^c^	-	0.03±0.01^a^	0.03±0.01^a^	0.18±0.04^ab^
J2	33.70±0.58^c^	0.03±0.01^a^	0.04±0.01^a^	0.03±0.01^a^	0.20±0.02^a^
J3	42.46±2.21^b^	0.02±0.01^ab^	0.05±0.01^a^	0.04±0.02^a^	0.17±0.01^ab^
J4	36.50±2.34^c^	0.02±0.01^ab^	0.04±0.01^a^	0.03±0.01^a^	0.14±0.02^b^
J5	46.30±5.23^b^	0.02±0.01^ab^	0.05±0.03^a^	0.04±0.01^a^	0.20±0.03^a^
J6	53.51±4.02^a^	0.02±0.01^b^	0.03±0.01^a^	0.02±0.01^a^	0.15±0.02^ab^

Note: The lowercase letters, CK, J1, J2, J3, J4, J5 and J6 are as in Table 1.

### 3.3. RNA-Seq analysis of jujube fruit

**Fig 2:**
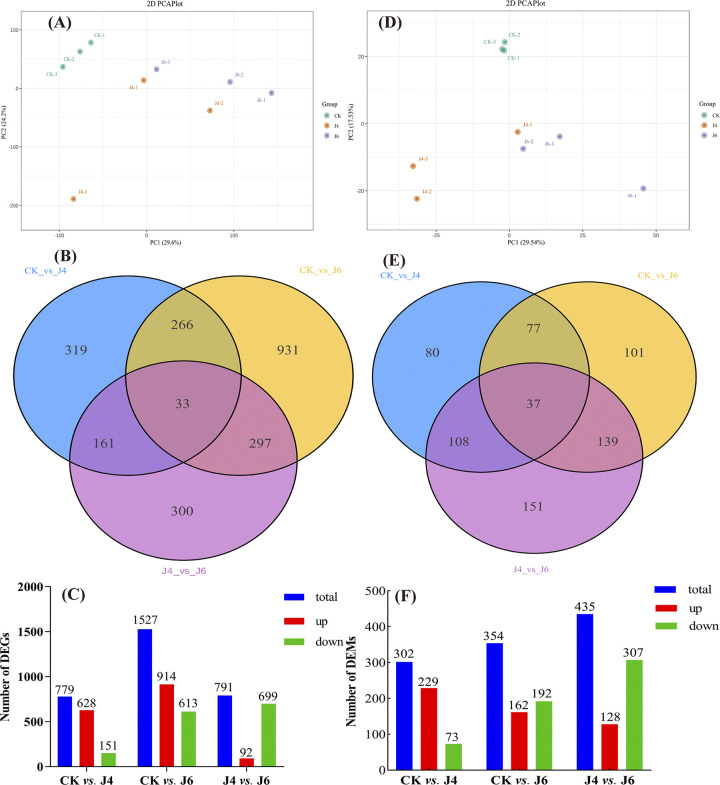
Transcriptomics and metabolomics analysis of jujube fruit treated with different plant growth regulator. (A) PCA analyses of RNA-Seq samples. (B) Venn diagram of DEGs between the control and treatment groups. (C) The number of up-regulated and down-regulated DEGs between the control and treatment groups. (D) PCA analyses of metabolomics samples. (E) Venn diagram of DEMs between the control and the treatment groups, (F) The number of up-regulated and down-regulated DEMs between the control and treatment groups. The meaning of CK, J4 and J6 are same as Fig 1.

**Fig 3:**
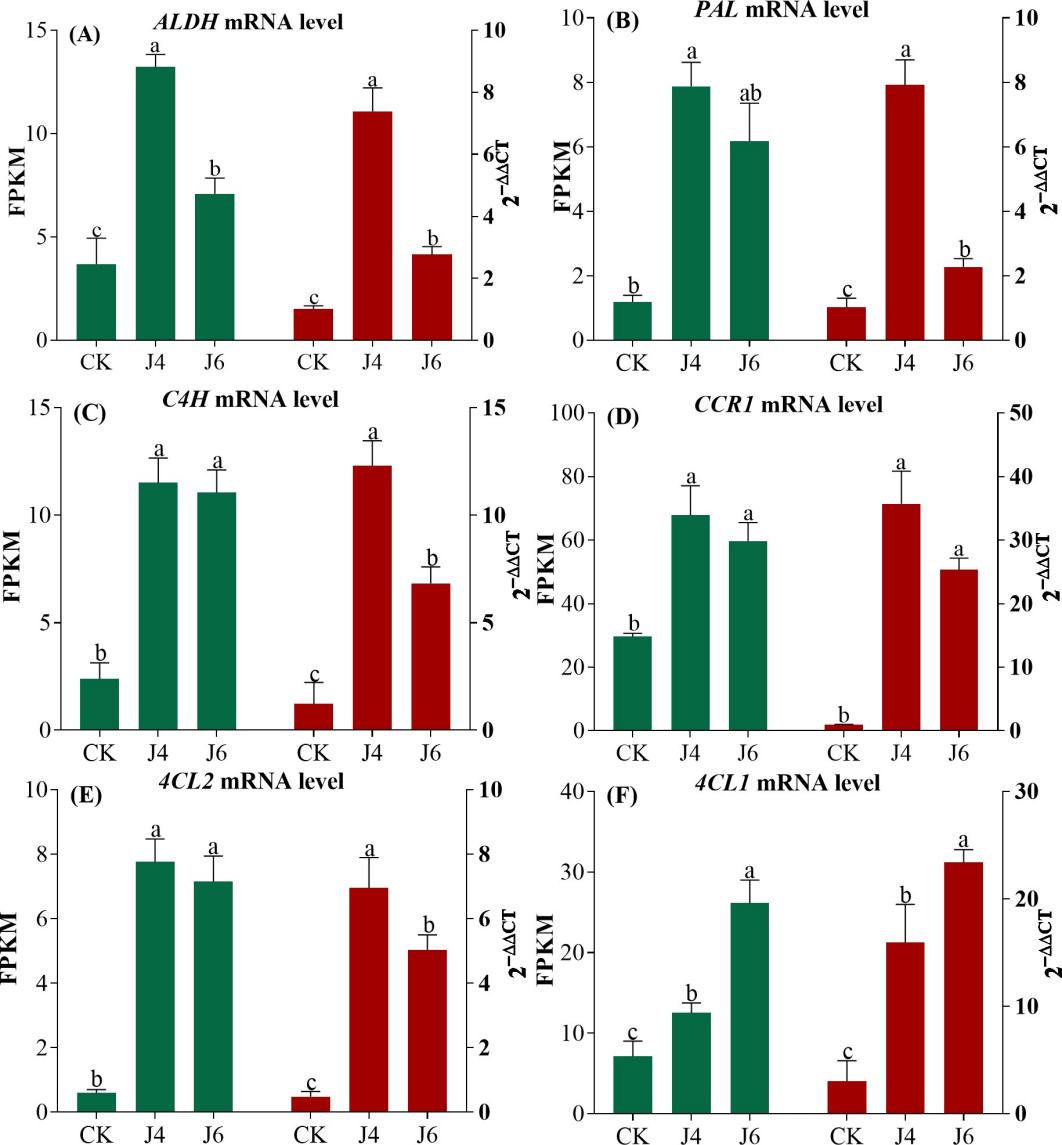
Plant growth regulator effects on phenylpropanoid pathway gene expression. (A-F) Relative expression levels of *ALDH*, *PAL*, *C4H*, *CCR1*, *4CL2*, *4CL1* from RNA-Seq and qRT-PCR, **respectively.** The meaning of CK, J4 and J6 are same as Fig 1.

A PCA analysis of fruit RNA-Seq data from CK and treatment groups were conducted. The PC1 and PC2 explained 29.6% and 24.2% variance, respectively, with a clear distinction between CK and treatment groups (Fig 2A). The correlation coefficient observed between samples of all treatments was greater than 0.89, and the correlation coefficient within treatment groups were greater than 0.93 (S1 Fig). These 779 DEGs (628 up-regulated and 151 down-regulated) were detected at J4, whereas 1527 (914 up-regulated and 613 down-regulated) were detected in fruit at J6. In addition, a comparison of J4 and J6 indicated 791 DEGs (92 up-regulated and 699 down-regulated) (Fig 2B and 2C). The biosynthetic pathway of phenylpropanoids promotes the accumulation of secondary metabolites (flavonoids and phenolic acids) in fruits. These mRNA levels of acetaldehyde dehydrogenase (*ALDH*), phenylalanine ammonia lyase (*PAL*), cinnamic acid 4-hydroxylase (*C4H*), Cinnamoyl-CoA reductase 1 (*CCR1*), 4-Coumarate Coenzyme A Ligase (*4CL2*, *4CL1*) were significantly increased at J4 and J6 (Fig 3). To confirm the validity of the RNA-Seq data, the relative expression levels of these phenylpropanoid pathway genes were measured by qRT-PCR. The relative expressions were approximately consistent with the FPKM value.

### 3.4. GO term and KEGG pathway analysis of jujube DEGs

**Fig 4:**
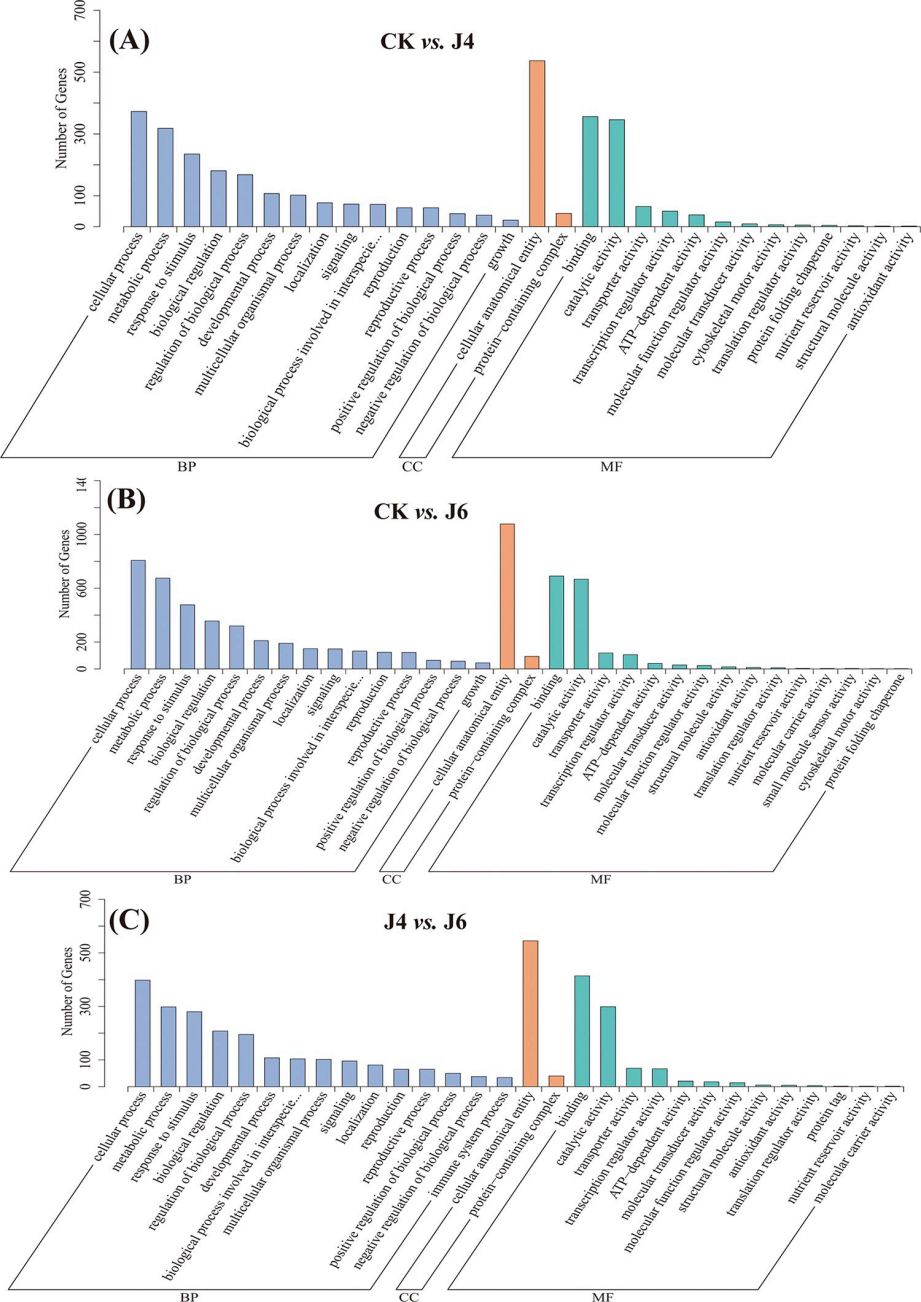
GO term analysis of DEGs in jujube fruit by application of plant growth regulator. (A) CK *vs*. J4. (B) CK *vs*. J6. (C) J4 *vs*. J6. The meaning of CK, J4 and J6 are same as Fig 1. The GO considered included cellular component (CC), biological process (BP) and molecular function (MF).

**Fig 5:**
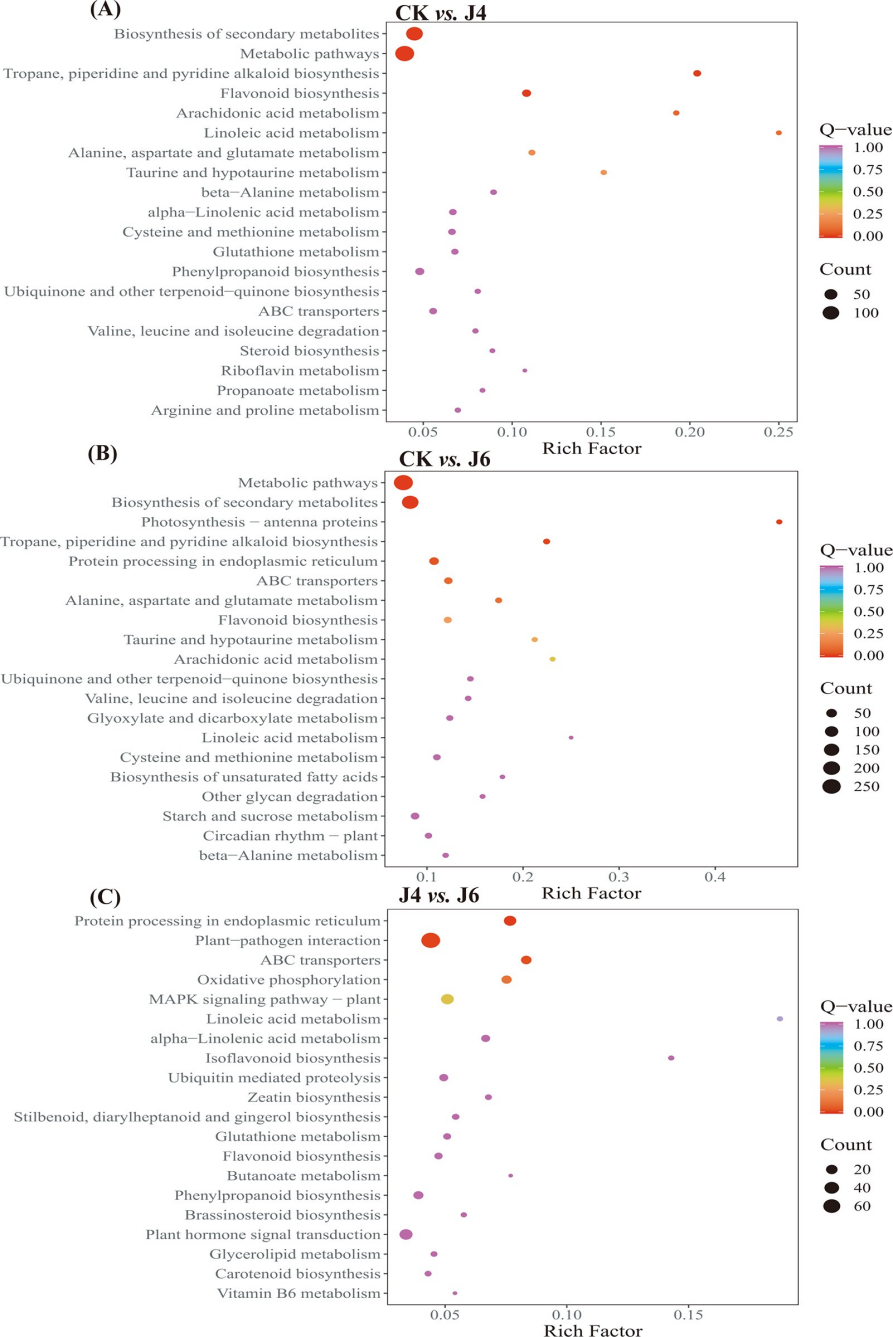
KEGG enrichment analysis of DEGs in jujube fruit by application of plant growth regulator. (A) CK *vs*. J4. (B) CK *vs*. J6. (C) J4 *vs*. J6. The meaning of CK, J4 and J6 are same as Fig 1.

An analysis of GO terms indicated that the DEGs affected at J4 and J6 were mainly enriched at cellular processes, metabolic processes, cellular anatomical entities, binding and catalytic activity (Fig 4). The top 20 of which for each pair-wise comparison are presented in Fig 5. Flavonoid biosynthetic and metabolic processes were enriched at J4 and J6 (Fig 5A and 5B). An examination of DEGs pertaining at J4 indicated 16 pertained to the phenylpropanoid biosynthesis pathway (ko00940; 14 up-regulated and 2 down-regulated), whereas the J6 resulted in 21 DEGs (17 up-regulated and 4 down-regulated). The six DEGs were selected for confirmation by qRT-PCR indicating that the RNA-Seq data was reliable (Fig 3).

### 3.5. Metabolomics analysis of different plant growth regulator effects in jujube fruits

**Fig 6:**
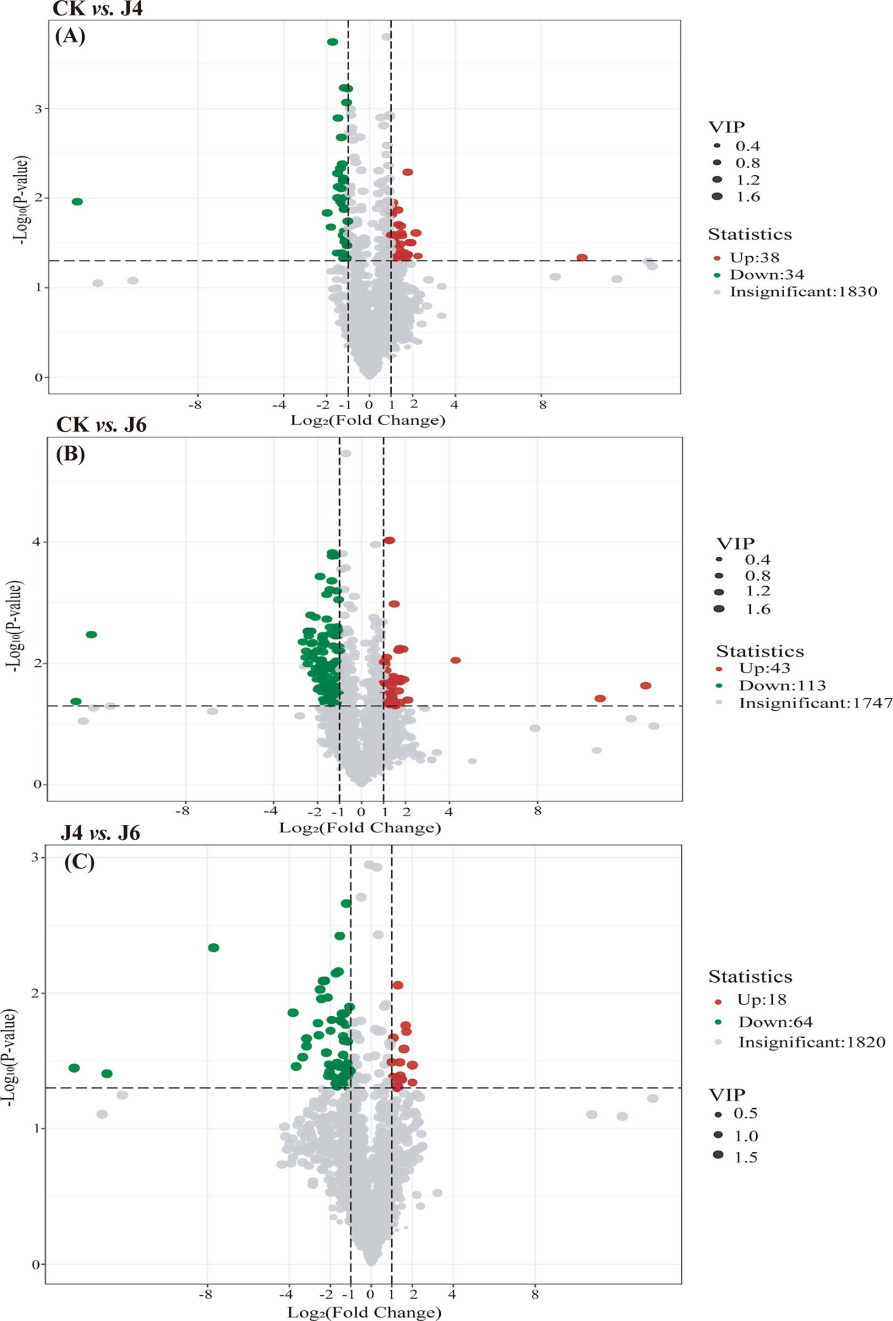
Volcano plots of DEMs in jujube fruit by application of plant growth regulator. (A) CK *vs*. J4. (B) CK *vs*. J6. (C) J4 *vs*. J6. The meaning of CK, J4 and J6 are same as Fig 1.

The metabolomic analysis of jujube fruit revealed the presence of 1091 DEMs between the control, J4 and J6. The PC1 and PC2 accounted for 32.1% and 17.7% of the total variance, respectively. The control group was clearly distinguished from the treatment groups by PC2, whereas the J4 and J6 could be distinguished by PC1, although two samples from each were proximal (Fig 2D). The orthogonal partial least squares-discriminant analysis (OPLS-DA) indicated 302 DEMs (229 up-regulated and 73 down-regulated) at J4, 354 DEMs (162 up-regulated and 192 down-regulated) at J6 and 435 DEMs (128 up-regulated and 307 down-regulated) between J4 *vs*. J6 (Fig 2E and 2F). The volcano plot of these DEMs obtained through further screening under VIP, log_2_ FC, P value screening conditions is shown in Fig 6A–6C.

**Fig 7:**
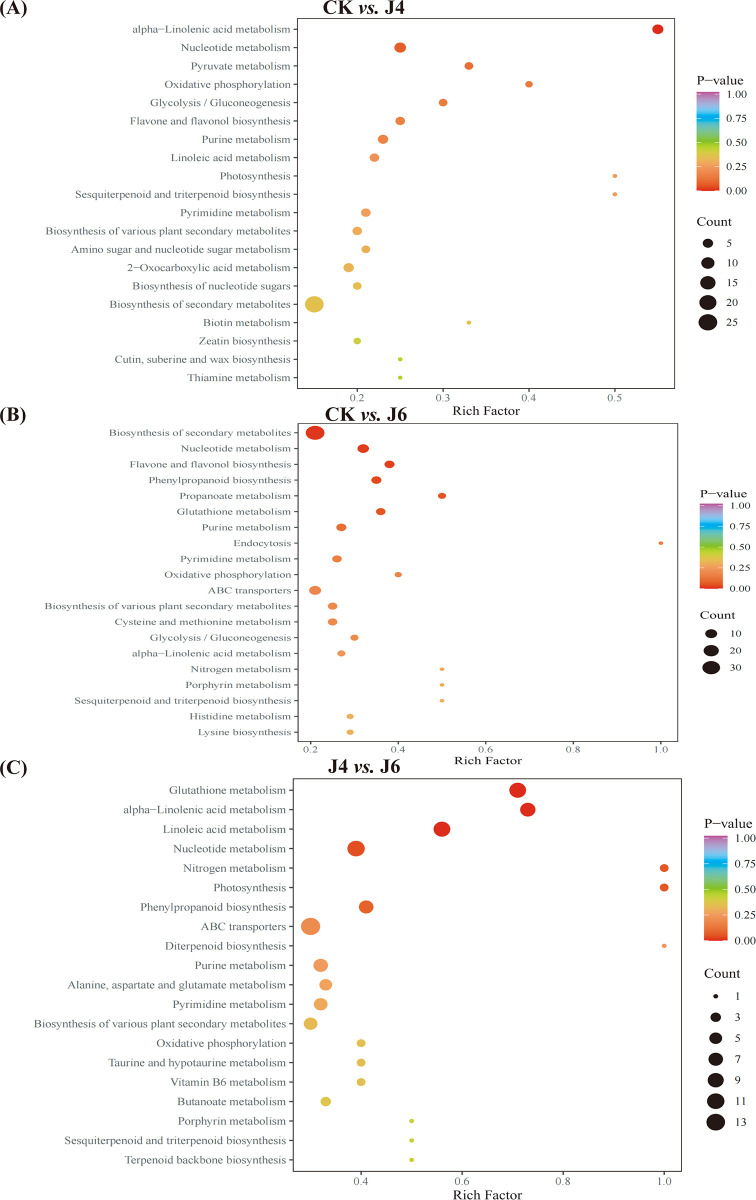
KEGG enrichment analysis of DEMs in jujube fruit by application of plant growth regulator. (A) CK *vs*. J4. (B) CK *vs*. J6. (C) J4 *vs*. J6. The meaning of CK, J4 and J6 are same as Fig 1.

To investigate the impact of plant growth regulator on different metabolic pathways, the DEMs identified were KEGG annotated (S2 Fig), followed by an enrichment analysis (Fig 6). Pathways enriched at J4 and J6 included nucleotide metabolism, flavone and flavonol biosynthesis and phenylpropanoid biosynthesis, although with differing levels of significance (Fig 7). KEGG pathway enrichment analysis of RNA-Seq data at J4 and J6 also indicated an enrichment of flavonoid biosynthesis and metabolism, with phenylpropanoid biosynthetic process at J4. Furthermore, fruit contents of lignin and flavonoids were increased at J4 and J6. Therefore, DEMs related with the phenylpropanoid pathway were examined further. The phenylpropanoid-related DEMs identified consisted of only cinnamic acid and sinapic acid at CK *vs*. J4; cinnamic acid, ferulic acid, syringin, caffeic aldehyde, 1-O-Sinapoyl-β-D-glucose and sinapyl alcohol at CK *vs*. J6.

### 3.6. Joint analysis of plant growth regulator effects on DEGs and DEMs related with the phenylpropanoid pathway in jujube fruit

A joint analysis of DEGs and DEMs enrichments related with the phenylpropanoid pathway were conducted to identify potential correlations (S3A–S3C Fig). These DEGs included the up-regulation of *PAL*, *C4H*, *4CL2*, *CCR*, Caffeoyl-CoA 3-O-methyltransferase (*CcoAoMT*) and the down-regulation of cinnamyl-alcohol dehydrogenase (*CAD*) at J4. These alterations were accompanied by significant increases cinnamic and sinapic acids (S3A Fig). These mRNA levels of *PAL*, *C4H*, *4CL*, *CCR* and coniferyl-alcohol glucosyltransferase (*CAGT*) were up-regulated and *CAD* was down-regulated at J6, which occurred with the higher accumulation of cinnamic acid, syringin, 1-O-Sinapoyl-β-D-glucose and sinapyl alcohol, but a reduction of ferulic acid and caffeic aldehyde (S3B Fig). These down-regulation of *CCR*, shikimate O-hydroxycinnamoyltransferase (*HCT*), *CAD*, peroxidase (*POD*) and *CcoAoMT*, and the up-regulation of *CAGT* occurred with increases in spermidine, syringin and sinapyl alcohol, with decreases in the levels of caffeic acid, sinapic acid, ferulic acid and caffeic aldehyde at J4 and J6 (C).

## 4. Discussion

GAs participates in various biological processes during fruit development [60]. The application of GA_3_ to persimmon trees has a significant impact on persimmon fruit size [61]. The treatment of green ripe bananas with cytokinin and GA_3_ significantly inhibited fruit softening and delayed ripening-related increases in respiration, ethylene content and soluble sugar accumulation, with a reduced loss of ascorbic acid and increases in total phenolic content [62]. In this study, all the plant growth regulator treatments utilized resulted in alterations to fruit phytohormone contents, including rapid, but short-term increases in GA_3_ and GA_1_ (2 h–1 d), and more prolonged changes, including relative increases in IAA and the suppression of ABA (Table 1).

The plant growth regulator treatments J4 and J6 significantly enhanced jujube fruit size. The fruit content of sucrose sugar in fruit was significantly increased by plant growth regulator treatments, which was consistent to that observed after similar treatment of grapevine, which was attributed to GA_3_ effects on organic acid conversion, sugar transport and accumulation [63]. Plant hormones play important roles in the regulation of phenylpropanoid biosynthesis [64,65]. GA treatment was reported to increase the phenolic content in wheat sprouts [66]. GA_3_ and cytokinin have a significant impact on the biosynthesis of phenylpropanoids and organic acids [67,68]. GA_3_ was shown to affect the synthesis of sugars and the regulation phenylpropanoid biosynthetic genes, thereby affecting the coloring and quality of grapes [68].

The outputs from the phenylpropanoid pathway includes flavonoids, phenolic compounds and lignin [69]. In this pathway, PAL is a key enzyme that catalyzes the deamination of phenylalanine to form cinnamic acid, which is then converted into *p*-coumaric acid by *C4H*, and *p*-coumaroyl-CoA by *4CL*. The *p*-coumaroyl-CoA is a major precursor metabolite of the flavonoid biosynthetic pathway, where enzymes such as 5*’* hydroxylase (*F3’5’H*) and flavonol synthase (*FLS1*) are involved in conversion steps leading to the formation of flavonoids such as quercetin, kaempferol and the various anthocyanins. The GA_3_ has been reported to increased PAL activity and the contents of phenols and flavonoids in long-on fruit [46]. The GA_3_ was also observed to increase flavonoid content in roselle [70]. The pear fruit with GAs also reduced lignin biosynthesis by down-regulating the expression of *PAL* and *4CL* [71].

In this study, we have shown that plant growth regulator treatments during jujube fruit development alter endogenous hormone levels, leading to alterations in the regulation of genes in aspects of the phenylpropanoid pathway (Figs 2B, 2C, 4 and 7), which is reflected in the contents of phenylpropanoid-related metabolites (Fig 2E and 2F). The phenylpropanoid pathway was directed to an increase in lignin deposition at J4 and J6 (Fig 1D). However, GO and KEGG analyses of RNA-Seq and metabolomics data also indicated an increased output from the flavonol/ flavonoid pathway (quercetin, epi-afzelechin, catechin, epicatechin, fustin) (Figs 4, 6 and 7). However, the specific regulatory mechanisms involved remain unclear.

## 5. Conclusions

Application of plant growth regulators increased the contents of GAs and IAA, but reduced the content of ABA. The 18 g·hm^−2^ GA_3_ is beneficial for increasing the soluble sugar and cAMP in jujube fruit, and reducing the organic acid. Both GA_3_ treatments increased lignin, but the lignin was lower in 18 g·hm^−2^ GA_3_. Therefore, it is recommended that 18 g·hm^−2^ GA_3_ during the flowering period of jujube. The combined analysis of RNA-Seq and metabolomics treated with different plant growth regulators indicated an up-regulation of *PAL*, *C4H*, *4CL2*, *CCR*, *CcoAoMT*, and the down-regulated *CAD* mRNA level of the phenylpropanoid pathway, which was accompanied by increased levels of cinnamic acid, eugenin, 1-O-sinapyl-D-glucose and sinapol, with decreases in ferulic and caffeic acids. These DEMs such as quercetin, epiafzelechin, catechin, epicatechin and fustin were increased at flavonoid pathway. Overall, our research findings have increased our understanding of how plant growth regulator affect quality of jujube fruit. In future, it would conduct in-depth research and validate the functional roles of key genes that control changes in flavonoids and polyphenols.

## Supporting information

S1 Fig Heat map of correlation coefficients between RNA-Seq samples. CK: the control group; J1: 18 g·hm^-2^ GA_3_; J2: 18 g·hm^-2^ GA_3_ and 45 mg·hm^-2^ BR; J3: 18 g·hm^-2^ GA_3_, 45 mg·hm^-2^ BR and 1.8 mg·hm^-2^ TDZ; J4: 36 g·hm^-2^ GA_3_; J5: 36 g·hm^-2^ GA_3_ and 45 mg·hm^-2^ BR; J6: 36 g·hm^-2^ GA_3_, 45 mg·hm^-2^ BR and 1.8 mg·hm^-2^ TDZ.
(TIF)

S2 Fig KEGG classification diagram of DEMs in jujube fruit. (A) CK *vs*. J4. (B) CK *vs*. J6. (C) J4 *vs*. J6. The meaning of CK, J4 and J6 are as in S1 Fig.
(TIF)

S3 Fig The plant growth regulator effects on the phenylpropanoid pathway from RNA-Seq and metabolomics. **The** red labels indicate the up-regulation of a gene/metabolite, while green labels indicate their down-regulation. (A) CK *vs*. J4. (B) CK *vs*. J6. (C) J4 *vs*. J6. The meaning of CK, J4 and J6 are as in S1 Fig.
(TIF)

S4 Fig TIC of volatile compounds in jujube fruit by GC-MS. (A) CK (B) J1 (C) J4. The meaning of CK, J1 and J4 are as in S1 Fig.
(TIF)

S1 Table Standard curves of plant hormone, sugar, organic acid, flavonoid and phenolic acid. (DOCX)

S2 Table UPLC-MS/MS conditions for the detection of GA_1_, GA_3_, GA_4_, GA_7_, IAA, ABA, flavone and polyphenols. (DOCX)

S3 Table Public databases used for the functional annotations of DEGs. (DOCX)

S4 Table Primer sequences utilized in qRT-PCR. (DOCX)

S5 Table Effects of different plant growth regulator formulations on the jujube fruit dimensions. (DOCX)

S6 Table The effect of GA_3_ on the types of volatile compounds in jujube. (DOCX)

S1 Graphical abstract (PNG)
