# Correction: Comprehensive analysis of transcriptomics and metabolomics provides insights into the mechanism by plant growth regulators affect the quality of jujube (*Ziziphus jujuba Mill.*) fruit

**DOI:** 10.1371/journal.pone.0327680

**Published:** 2025-07-02

**Authors:** The PLOS One Staff

There are errors in the author affiliations. The correct affiliations are as follows:

Defen Liu^1,2^, Na Jiang^1^,Yuting Yuan^1,2^, Hejiang Liu^1^, Yanjun Ju^1^,WanjinSun^3^, Wenzhao Jia^4^, Yi Fang^4^, DuoyongZhao^1^, Jiefei Mao^5^, LuKang^1,5.^

**1** Xinjiang Academy of Agricultural Sciences Institute of Agricultural Quality Standards and Testing Technology/Xinjiang Key Laboratory of Agricultural Product Quality and Safety, Urumqi, China, **2** College of Food Science and Pharmacy, Xinjiang Agricultural University, Urumqi, China, **3** Social Affairs Service Center of the Eighth Regiment of the First Division of Xinjiang Production and Construction Corps, Alar, China, **4** Agricultural Development Service Center of the Eighth Regiment of the First Division of Xinjiang Production and Construction Corps, Alar, China, **5** Key Laboratory of Ecological Safety and Sustainable Development in Arid Lands, Xinjiang Institute of Ecology and Geography, State Key Laboratory of Desert and Oasis Ecology, Chinese Academy of Sciences, Urumqi, China

The publisher apologizes for the errors.
