# Supplementary figures and images for: Comprehensive analysis of transcriptomics and metabolomics provides insights into the mechanism by plant growth regulators affect the quality of jujube (Ziziphus jujuba Mill.) fruit
Source: PLoS One. 2024 Aug 23;19(8):e0305185. doi: 10.1371/journal.pone.0305185 (PMC11343422; doi:10.1371/journal.pone.0305185)

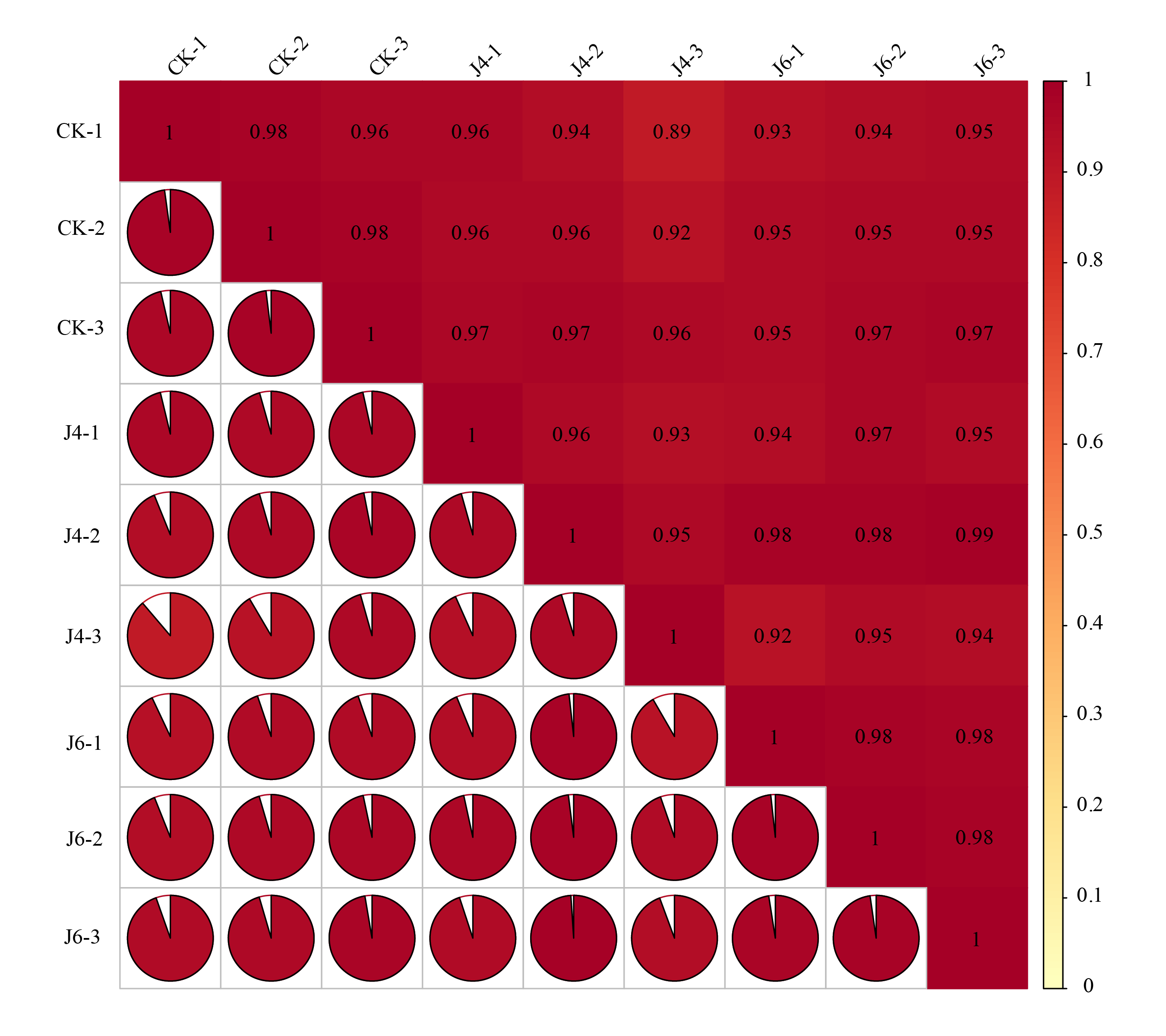

Supplement: S1 Fig — CK: the control group; J1: 18 g·hm-2 GA3; J2: 18 g·hm-2 GA3 and 45 mg·hm-2 BR; J3: 18 g·hm-2 GA3, 45 mg·hm-2 BR and 1.8 mg·hm-2 TDZ; J4: 36 g·hm-2 GA3; J5: 36 g·hm-2 GA3 and 45 mg·hm-2 BR; J6: 36 g·hm-2 GA3, 45 mg·hm-2 BR and 1.8 mg·hm-2 TDZ. (TIF) [file pone.0305185.s001.tif]

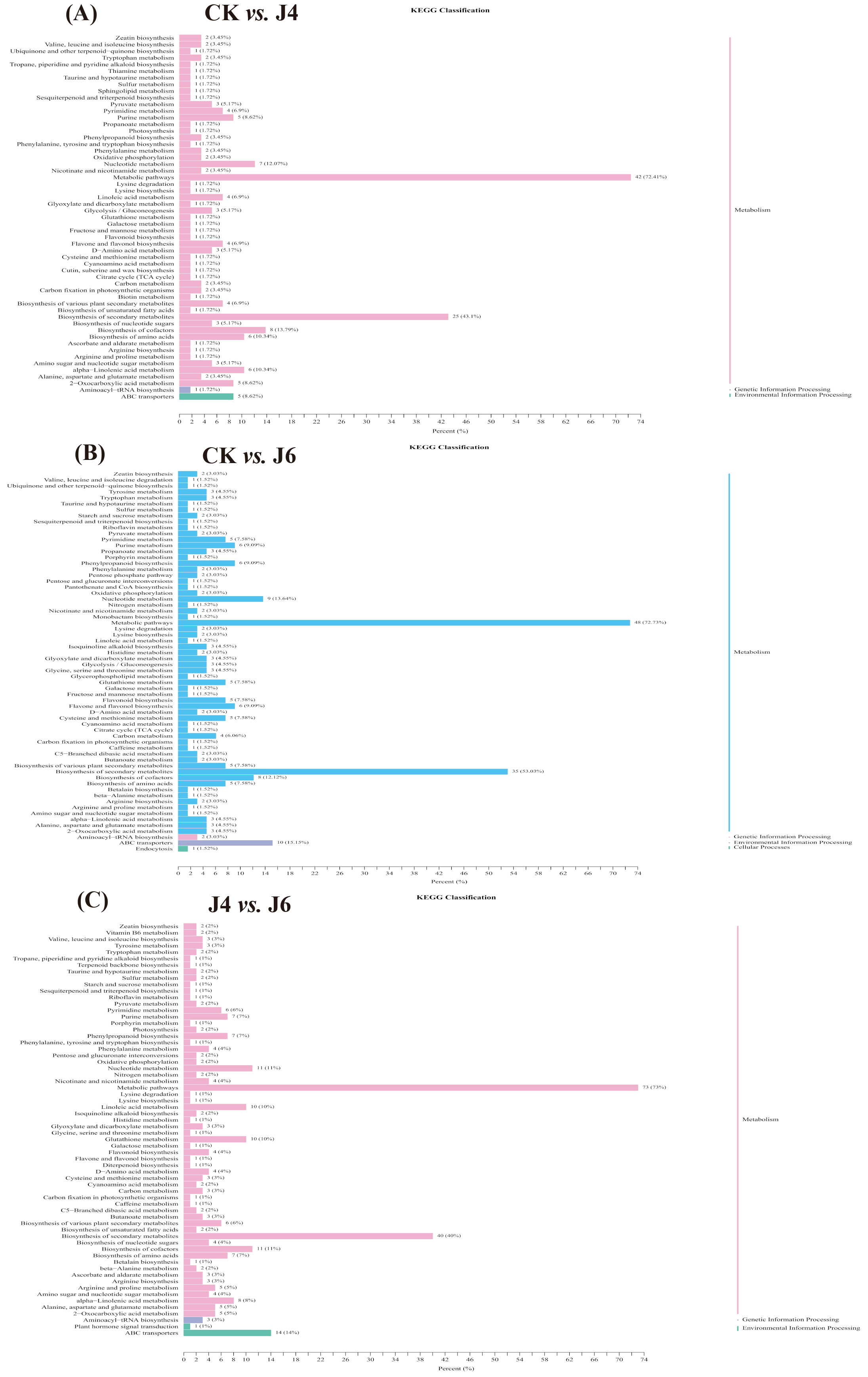

Supplement: S2 Fig — (A) CK vs. J4. (B) CK vs. J6. (C) J4 vs. J6. The meaning of CK, J4 and J6 are as in S1 Fig. (TIF) [file pone.0305185.s002.tif]

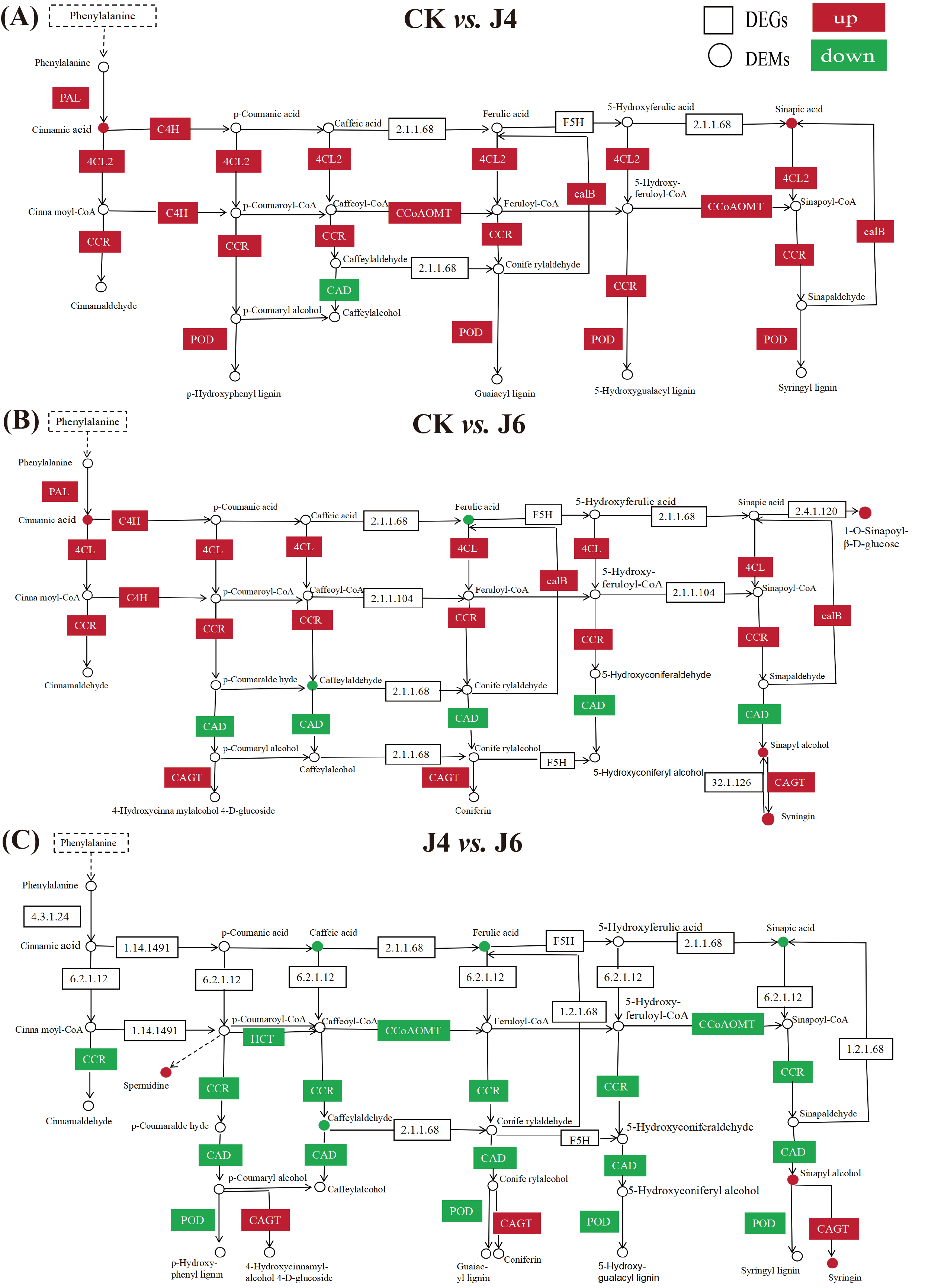

Supplement: S3 Fig — The red labels indicate the up-regulation of a gene/metabolite, while green labels indicate their down-regulation. (A) CK vs. J4. (B) CK vs. J6. (C) J4 vs. J6. The meaning of CK, J4 and J6 are as in S1 Fig. (TIF) [file pone.0305185.s003.tif]

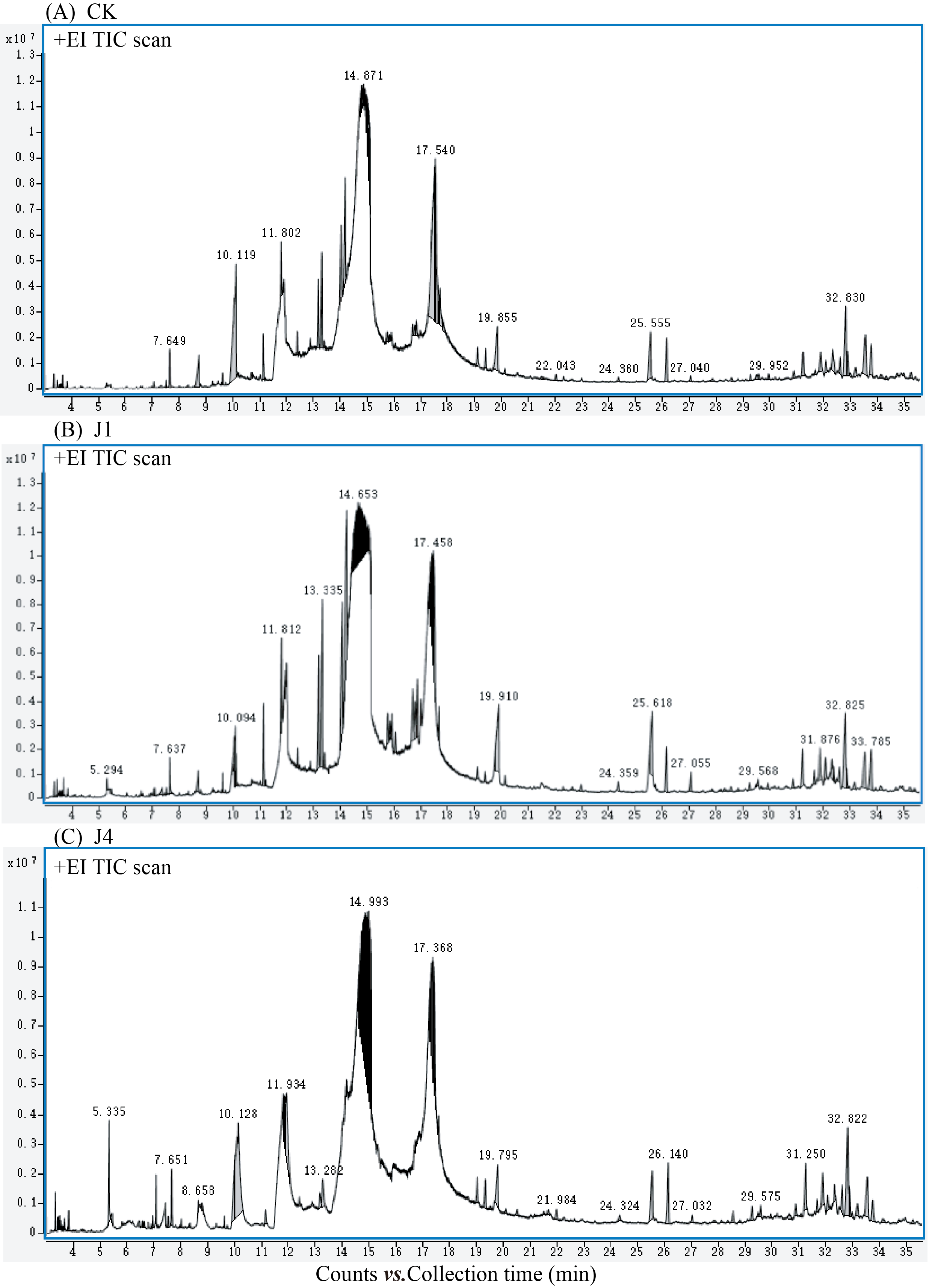

Supplement: S4 Fig — (A) CK (B) J1 (C) J4. The meaning of CK, J1 and J4 are as in S1 Fig. (TIF) [file pone.0305185.s004.tif]

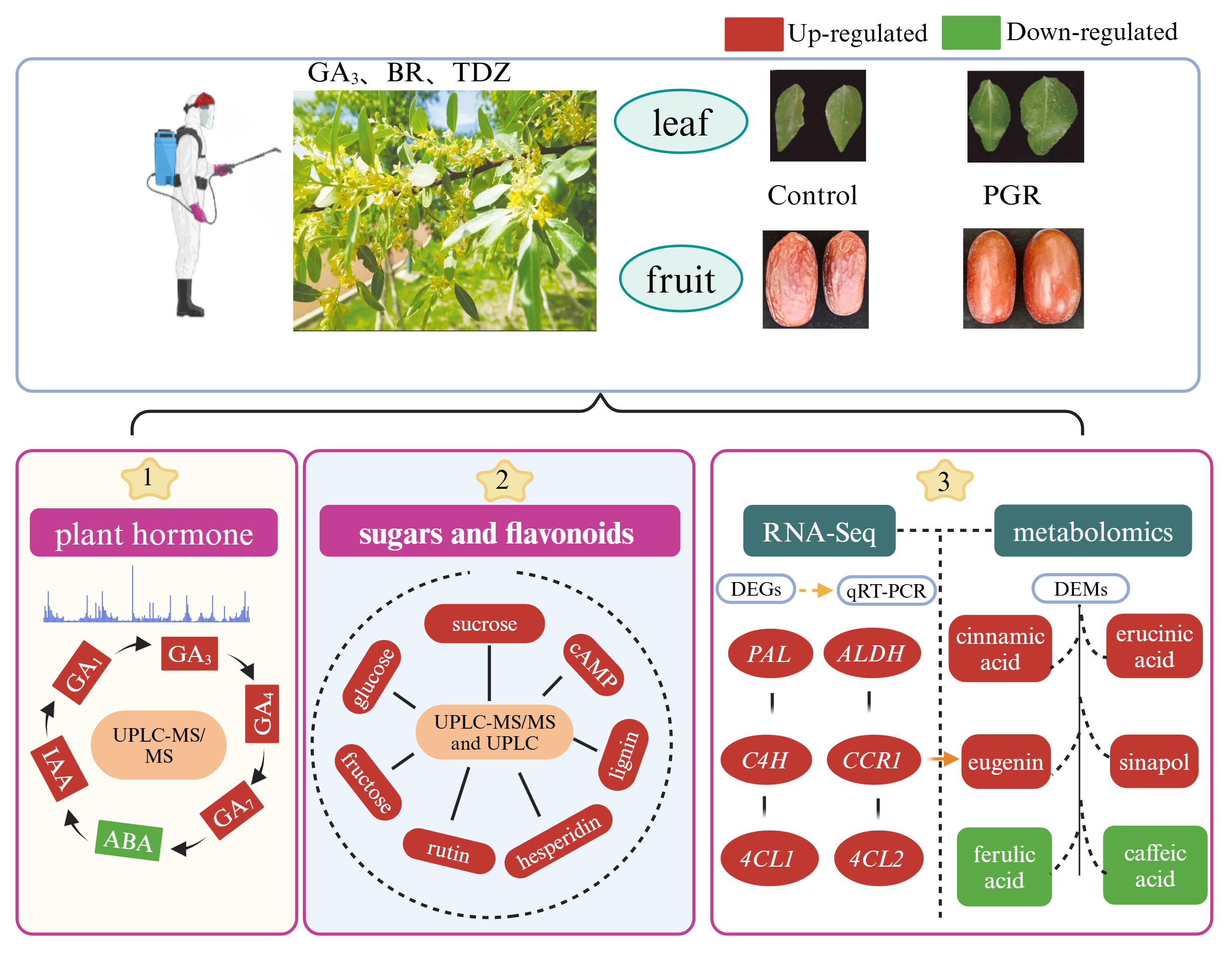

Supplement: S1 Graphical abstract — (PNG) [file pone.0305185.s011.png]
