# Supplementary material for: Comprehensive analysis of transcriptomics and metabolomics provides insights into the mechanism by plant growth regulators affect the quality of jujube (Ziziphus jujuba Mill.) fruit
Source: PLoS One. 2024 Aug 23;19(8):e0305185. doi: 10.1371/journal.pone.0305185 (PMC11343422; doi:10.1371/journal.pone.0305185)
Supplement: S1 Table — (DOCX) [file pone.0305185.s005.docx]

Table S1. Standard curves of plant hormone, sugar, organic acid, flavonoid and phenolic acid

| **Component** | **Purity** | CAS **number** | **Equation** | R |
| --- | --- | --- | --- | --- |
| GA1 | 97.10% | 545-97-1 | y=3.5863x-32.81 | 0.9994 |
| GA3 | 97.30% | 77-06-5 | y=90.245x-1187.5 | 0.9993 |
| GA4 | 97.50% | 468-44-0 | y=38.735x-430.95 | 0.9992 |
| GA7 | 97.10% | 510-75-8 | y=45.874x-366.38 | 0.9994 |
| IAA | 96.10% | 87-51-4 | y=9.2373x-139.48 | 0.9991 |
| ABA | 99.80% | 14375-45-2 | y=368.15x-4053.4 | 0.9991 |
| Fructose | 99.90% | 57-48-7 | y=149153x+189971 | 0.999 |
| Glucose | 99.80% | 50-99-7 | y=100030x+127030 | 0.9996 |
| Sucrose | 99.80% | 57-50-1 | y=100030x+127030 | 0.9996 |
| Malic acid | 99.70% | 6915-15-7 | y=459.1x+47173 | 0.9999 |
| Tartaric acid | 99.70% | 133-37-9 | y=673.92x+55625 | 0.9991 |
| Citric acid | 99.20% | 5949-29-1 | y=632.48x+39922 | 0.9998 |
| Fumaric acid | 99.50% | 110-17-8 | y=115162x+21167 | 0.9999 |
| cAMP | 97.10% | 60-92-4 | y=19719x-10963 | 0.9991 |
| Hesperidin | 96.10% | 520-26-3 | y=0.3145x+7.2067 | 0.9995 |
| Catechin | 93.20% | 154-23-4 | y=1.0876x+17.371 | 0.9998 |
| Rutin | 86.80% | 153-18-4 | y=0.1023x-0.3161 | 0.9996 |
| Naringin | 89.70% | 10236-47-2 | y=4.5295x+146.6 | 0.999 |
| Kaempferol | 89.70% | 520-18-3 | y=1.0609x+38.426 | 0.999 |
| Quercetin | 95.30% | 117-39-5 | y=1.8053x+26.903 | 0.9998 |
| Quinic acid | 91.70% | 77-95-2 | y=3.0633x+32.541 | 0.9998 |
| Chlorogenic acid | 93.70% | 327-97-9 | y=4.8983x+126.42 | 0.999 |
| Caffeic acid | 99.70% | 331-39-5 | y=33.448x+1216.4 | 0.9991 |
| Ferulic | 97.30% | 1135-24-6 | y=10.523x+232.28 | 0.9996 |
| Trans-4-hydroxy-cinnamic acid | 97.30% | 501-98-4 | y=70.001x+761.61 | 0.9991 |

GA_1_: gibberellin A1, GA_3_: gibberellin A3, GA_4_: gibberellin A4, GA_7_: gibberellin A7, IAA: indole-3-acetic acid, ABA: abscisic acid, cAMP: cyclic adenosine monophosphate.
