# Supplementary material for: Comprehensive analysis of transcriptomics and metabolomics provides insights into the mechanism by plant growth regulators affect the quality of jujube (Ziziphus jujuba Mill.) fruit
Source: PLoS One. 2024 Aug 23;19(8):e0305185. doi: 10.1371/journal.pone.0305185 (PMC11343422; doi:10.1371/journal.pone.0305185)
Supplement: S2 Table — (DOCX) [file pone.0305185.s006.docx]

Table S2. UPLC-MS/MS conditions for the detection of GA_1_, GA_3_, GA_4_, GA_7_, IAA, ABA, flavone and polyphenols

| Name | Formula | Parent Ion | Daughter Ion | Cone(V) | Collision(V) |
| --- | --- | --- | --- | --- | --- |
| GA_1_ | C_19_H_22_O_6_ | 347.2 | 241.2 | 74 | 15 |
|  |  |  | 259.3 | 74 | 12 |
| GA_3_ | C_19_H_22_O_6_ | 345.2 | 143.3 | 18 | 27 |
|  |  |  | 239.3 | 18 | 16 |
| GA_4_ | C_19_H_24_O_5_ | 331.2 | 213.3 | 71 | 25 |
|  |  |  | 225.3 | 71 | 14 |
| GA_7_ | C_19_H_22_O_5_ | 331.2 | 225.3 | 21 | 23 |
|  |  |  | 257.3 | 21 | 24 |
| IAA | C₁₀H₉NO₂ | 174.2 | 128.0 | 22 | 20 |
|  |  |  | 130.1 | 22 | 9 |
| ABA | C_15_H_20_O_4_ | 263.1 | 153.1 | 26 | 15 |
|  |  |  | 219.1 | 26 | 11 |
| Hesperidin | C_28_H_34_O_15_ | 609.0 | 301.1 | 48 | 26 |
|  |  |  | 164.0 | 48 | 56 |
| catechin | C_15_H_14_O_6_ | 297.2 | 154.3 | 22 | 20 |
|  |  |  | 98.9 | 22 | 17 |
| Rutin | C_27_H_30_O_16_ | 609.1 | 300.0 | 60 | 36 |
|  |  |  | 300.0 | 60 | 58 |
| Naringenin | C_15_H_12_O_5_ | 270.9 | 119.1 | 8 | 18 |
|  |  |  | 151.0 | 8 | 26 |
| Kaempferol | C_15_H_10_O_6_ | 284.8 | 93.0 | 50 | 20 |
|  |  |  | 151.0 | 50 | 26 |
| Quercitrin | C_21_H_20_O_11_ | 446.9 | 300.2 | 54 | 28 |
|  |  |  | 271.1 | 54 | 40 |
| Quinic acid | C_7_H_12_O_6_ | 190.9 | 85.0 | 24 | 20 |
|  |  |  | 93.1 | 24 | 16 |
| Chlorogenic acid | C_16_H_18_O_9_ | 352.9 | 191.1 | 12 | 18 |
|  |  |  | 85.0 | 12 | 38 |
| Caffeic acid | C_9_H_8_O_4_ | 178.9 | 135.0 | 22 | 15 |
|  |  |  | 79.0 | 22 | 24 |
| Ferulic acid | C_10_H_10_O_4_ | 192.9 | 134.1 | 21 | 15 |
|  |  |  | 178.1 | 21 | 12 |
| Trans-4-hydroxy-  cinnamic acid | C_9_H_8_O_3_ | 163.0 | 118.9 | 9 | 19 |
|  |  |  | 91.1 | 9 | 9 |
