# Supplementary material for: Comprehensive analysis of transcriptomics and metabolomics provides insights into the mechanism by plant growth regulators affect the quality of jujube (Ziziphus jujuba Mill.) fruit
Source: PLoS One. 2024 Aug 23;19(8):e0305185. doi: 10.1371/journal.pone.0305185 (PMC11343422; doi:10.1371/journal.pone.0305185)
Supplement: S3 Table — (DOCX) [file pone.0305185.s007.docx]

Table S3. Public databases used for the functional annotations of DEGs

| Database | Annotated number |
| --- | --- |
| KEGG | 17,391 |
| KOG | 28,061 |
| NR | 28,194 |
| Pfam | 24,319 |
| Swissprot | 21,954 |
| Tremble | 28,033 |
| GO | 24,314 |
