# Supplementary material for: Comprehensive analysis of transcriptomics and metabolomics provides insights into the mechanism by plant growth regulators affect the quality of jujube (Ziziphus jujuba Mill.) fruit
Source: PLoS One. 2024 Aug 23;19(8):e0305185. doi: 10.1371/journal.pone.0305185 (PMC11343422; doi:10.1371/journal.pone.0305185)
Supplement: S4 Table — (DOCX) [file pone.0305185.s008.docx]

Table S4. Primer sequences utilized in qRT-PCR

| Gene ID | Gene Name | Forward primer (5' to 3') | Reverse primer (5' to 3') | Product size |
| --- | --- | --- | --- | --- |
| LOC107431863 | *ALDH* | AGGAGGCGATACAGAGAGCA | CCATAAGGGCAGTCTCGGTC | 157 |
| LOC107404213  LOC107413687 | *PAL* | CGGGGTTTTTCGAGTTGCAG | ATTGCTGCGGCTTCGATTTG | 226 |
| LOC107406287 | *C4H* | TGATGCCCAGCAGAAAGGAG | CTCGGTGATCGGATTCCCTG | 196 |
| LOC107409091  LOC107416048 | *CCR1* | AGCAGCCGAAGCCAAAGTTA | GCCACTGCCTTACCATAGCA | 165 |
| LOC107414888 | *4CL2* | GCCCTTCTCATCGGGAACAA | ATCAAAACCGCTGCACCAAC | 213 |
| LOC107413992 | *4CLL1* | TTCGCCAAACAAAACGGCAT | GATTGATTTTCACCGCCGGG | 133 |
| LOC107413530 | *ACT1* | AGCCTTCCTGCCAACGAGT | TTGCTTCTCACCCTTGATGC | 125 |
