# Supplementary material for: Comprehensive analysis of transcriptomics and metabolomics provides insights into the mechanism by plant growth regulators affect the quality of jujube (Ziziphus jujuba Mill.) fruit
Source: PLoS One. 2024 Aug 23;19(8):e0305185. doi: 10.1371/journal.pone.0305185 (PMC11343422; doi:10.1371/journal.pone.0305185)
Supplement: S5 Table — (DOCX) [file pone.0305185.s009.docx]

Table S5. Effects of different plant growth regulator formulations on the jujube fruit dimensions (cm)

| Item | Different treatment groups (mean ± standard deviation) | | | | | | |
| --- | --- | --- | --- | --- | --- | --- | --- |
|  | CK | J1 | J2 | J3 | J4 | J5 | J6 |
| Transverse diameter | 4.23±0.40c | 4.97±0.12b | 4.40±0.17c | 5.13±0.06a | 5.23±0.12b | 5.03±0.28b | 5.63±0.23a |
| Longitudinal diameter | 2.90±0.17b | 3.03±0.23b | 3.01±0.09b | 2.93±0.05b | 3.37±0.12a | 3.07±0.05b | 3.06±0.06b |

Note: Different lowercase letters in each row indicate significant differences (*P*<0.05) between the plant growth regulator treatments. CK: the control group; J1: 18 g·hm^-2^ GA_3_; J2: 18 g·hm^-2^ GA_3_ and 45 mg·hm^-2^ BR; J3: 18 g·hm^-2^ GA_3_, 45 mg·hm^-2^ BR and 1.8 mg·hm^-2^ TDZ; J4: 36 g·hm^-2^ GA_3_; J5: 36 g·hm^-2^ GA_3_ and 45 mg·hm^-2^ BR; J6: 36 g·hm^-2^ GA_3_, 45 mg·hm^-2^ BR and 1.8 mg·hm^-2^ TDZ.
