# Supplementary material for: Comprehensive analysis of transcriptomics and metabolomics provides insights into the mechanism by plant growth regulators affect the quality of jujube (Ziziphus jujuba Mill.) fruit
Source: PLoS One. 2024 Aug 23;19(8):e0305185. doi: 10.1371/journal.pone.0305185 (PMC11343422; doi:10.1371/journal.pone.0305185)
Supplement: S6 Table — (DOCX) [file pone.0305185.s010.docx]

Table S6. The effect of GA_3_ on the types of volatile compounds in jujube

| Type | CK | J1 | J4 |
| --- | --- | --- | --- |
| Acids | 15 | 20 | 25 |
| Esters | 13 | 12 | 9 |
| Alcohols | 11 | 7 | 7 |
| Ketones | 7 | 6 | 7 |
| Ethers | ND | ND | 1 |
| Aldehydes | 1 | 1 | ND |

Note: The meaning of CK, J1 and J4 are as table S5. ND indicates not detected.
